# The Quality of Indian Obesity-Related mHealth Apps: PRECEDE-PROCEED Model–Based Content Analysis

**DOI:** 10.2196/15719

**Published:** 2022-05-11

**Authors:** Shanmuga Nathan Selvaraj, Arulchelvan Sriram

**Affiliations:** 1 Department of Media Sciences College of Engineering Anna University Chennai India

**Keywords:** obesity, mHealth apps, PRECEDE-PROCEED Model, Mobile App Rating Scale, health communication, health behavior change techniques, health information

## Abstract

**Background:**

The prevalence of obesity in India is increasing at an alarming rate. Obesity-related mHealth apps have proffered an exciting opportunity to remotely deliver obesity-related information. This opportunity raises the question of whether such apps are truly effective.

**Objective:**

The aim of this study was to identify existing obesity-related mHealth apps in India and evaluate the potential of the apps’ contents to promote health behavior change. This study also aimed to discover the general quality of obesity-related mHealth apps.

**Methods:**

A systematic search for obesity-related mHealth apps was conducted in both the Google Play Store and the Apple App Store. The features and quality of the sample apps were assessed using the Mobile Application Rating Scale (MARS) and the potential of the sample apps’ contents to promote health behavior change was assessed using the PRECEDE-PROCEED Model (PPM).

**Results:**

A total of 13 apps (11 from the Google Play Store and 2 from the Apple App Store) were considered eligible for the study. The general quality of the 13 apps assessed using MARS resulted in mean scores ranging from 1.8 to 3.7. The bivariate Pearson correlation between the MARS rating and app user rating failed to establish statistically significant results. The multivariate regression analysis result indicated that the PPM factors are significant determinants of health behavior change (*F*_3,9_=63.186; *P*<.001) and 95.5% of the variance (*R*^2^=0.955; *P*<.001) in the dependent variable (health behavior change) can be explained by the independent variables (PPM factors).

**Conclusions:**

In general, mHealth apps are found to be more effective when they are based on theory. The presence of PPM factors in an mHealth app can greatly influence the likelihood of health behavior change among users. So, we suggest mHealth app developers consider this to develop efficient apps. Also, mHealth app developers should consider providing health information from credible sources and indicating the sources of the information, which will increase the perceived credibility of the apps among the users. We strongly recommend health professionals and health organizations be involved in the development of mHealth apps. Future research should include mHealth app users to understand better the apps’ effectiveness in bringing about health behavior change.

## Introduction

### Background

Obesity is an alarming health issue that leads to significant health and social difficulties for people globally. Generally, obesity is defined by the measurement of the BMI [1]. Per clinical guidelines, a BMI of 25 kg/m^2^ to 29.9 kg/m^2^ indicates overweight or preobesity and a BMI of 30 kg/m^2^ or greater indicates obesity [2]. Obesity is associated with all-cause mortality. The health consequences of obesity are vast, including cardiovascular diseases, diabetes, musculoskeletal disorders, and some cancers, such as endometrial, breast, and colon cancer. The next generations are in a more dangerous position since the health consequences of childhood obesity are extensive, including premature death and disability in adulthood [3].

### Obesity in India

An increase in the consumption of junk food and the adoption of sedentary lifestyles are the major reasons for the increase in the prevalence of obesity in India. According to the India National Family Health Survey-4, the number of people with obesity in India doubled between 2006 and 2016. The prevalence of obesity among women ages 5 to 49 years in India is 20.7%, which is a 60% increase from 2005 to 2006. The prevalence of obesity among men ages 5 to 49 years in India doubled to 18.6% from 9.3% in the year 2005 to 2006 [4,5]. A study involving 14.4 million children in India revealed that the country has the second-highest prevalence of childhood obesity in the world after China [3]. The prevalence of obesity in India is increasing at an alarming rate.

### Obesity and Media

Obesity is the fastest-growing global public health issue and media campaigns can increase public awareness of obesity [6]. Media campaigns are found to be more effective in raising awareness about the causes of obesity, health problems associated with obesity, and healthy habits to prevent and manage obesity [7,8]. Public attention to a particular issue correlates with the degree of salience of the issues covered in the media. Media can be used to provide information as simply as possible and to update the information constantly [9]. Though media can have an impact on knowledge and attitudes about obesity among the public, evidence is still limited as to whether media can influence health behavior change [10].

### mHealth Apps for Obesity

Television was the dominant form of media for increasing obesity awareness, but with the rapid advance of digital media, the evaluation of other media, such as internet-based media, is increasingly important [10]. The most recent and fastest evolving internet-based media is mobile media [11]. Substantially, mobile media are used for the delivery of health information [12]. The World Health Organization defined mHealth as medical and public health practices supported by mobile devices [13]. Smartphones have gained popularity and are being adopted for mHealth practices. There are different types of mHealth apps developed and available for general use in obesity management [14]. The benefits of mHealth apps include cost-effectiveness, the potential for real-time data collection, feedback capability, minimized participant burden, relevance to multiple populations, and increased dissemination capability [15]. Obesity-related mHealth apps have proffered an exciting opportunity to remotely deliver obesity-related information. This opportunity raises the question of whether such apps are truly effective. Therefore, the purpose of this study was to identify existing obesity-related mHealth apps in India and evaluate the potential of the app contents in promoting health behavior change.

### The PRECEDE-PROCEED Model

The PRECEDE-PROCEED Model (PPM) is a widely accepted health education framework for planning and evaluating health behavior change programs [16,17]. The anticipated influence on health behavior change can be evaluated by the presence of 3 factors in health interventions, predisposing factors, enabling factors, and reinforcing factors. Predisposing factors include the following variables, which act as antecedents to health behavior change: knowledge, attitudes, beliefs, values, and motivation. Enabling factors include the following variables, which act as antecedents that facilitate health behavior change: teaching skills, providing resources, providing a service, and tracking progress. Reinforcing factors include the following variables, which provide rewards or feedback for health behavior change: interacting with health professionals to obtain support and interfacing with social media sites for encouragement [18]. This study attempts to identify the presence of PPM variables in Indian obesity-related mHealth apps for promoting health behavior change. This study also aimed to examine the overall quality of obesity-related mHealth apps.

## Methods

This study involved a qualitative content analysis of the available obesity-related mHealth apps in the Google Play Store and Apple App Store.

### Study Sample

There are studies showing that mHealth app users are more likely to use free apps, which is why most previous studies on mHealth apps focused only on free apps [19] (R Subramanian, PhD, unpublished data, August 2015). Likewise, this study will focus only on free obesity-related mHealth apps. Free obesity-related apps were identified using the following search terms in the Google Play Store and Apple App Store during June 2021: “obesity”, “obese”, “obesity calculator”, “obesity diet”, and “obesity exercise”. An app was considered for inclusion if the app content had obesity related-information and the app was rated above 3 out of 5 stars.

### Measurement

**Figure 1:**
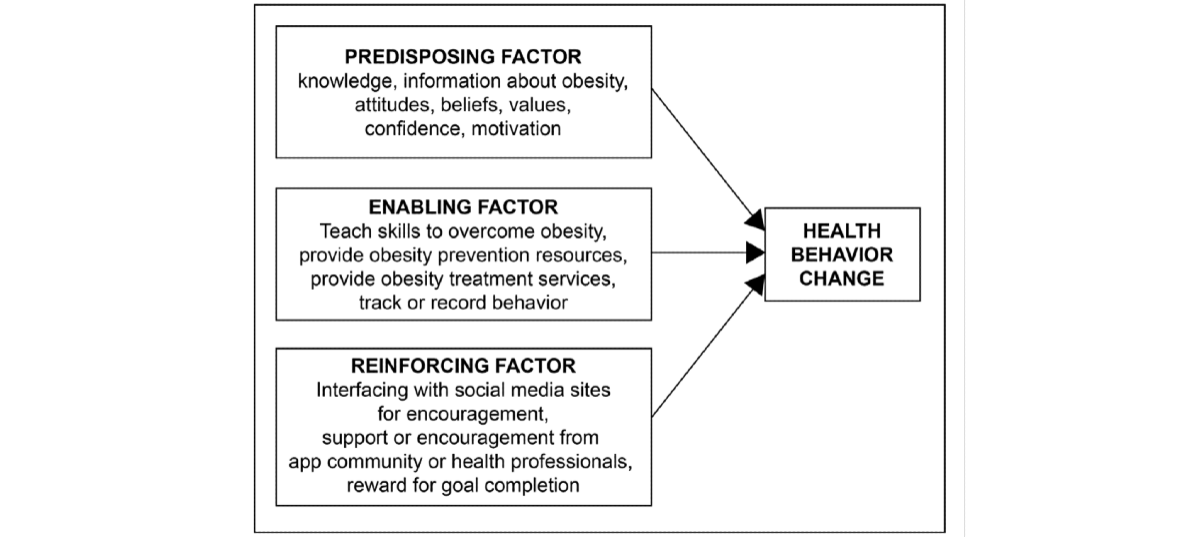
Framework of PRECEDE-PROCEED Model factors influencing health behaviour change [23].

Each sample app was coded for basic descriptive information, such as the app name, user rating, and the number of downloads. The features and quality of the sample apps were assessed using the Mobile Application Rating Scale (MARS) [20-22] and the potential of the app contents to promote health behavior change was assessed using the PPM [18]. MARS is a measure for classifying and assessing the quality of mHealth apps. The MARS uses a Likert scale ranging from 1 (inadequate) to 5 (excellent) to score apps on the following criteria: engagement, functionality, aesthetics, information quality, and subjective quality [22]. The PPM (Figure 1) was used to measure each app according to its level of anticipated influence on health behavior change.

### Data Collection

The MARS and PPM were explained to 2 coders, who were researchers studying mHealth apps with several years of experience and a good knowledge of mHealth apps [24,25]. The coding sheet is presented in Multimedia Appendix 1. The coders were instructed on each measure and its definition to ensure clear differentiation between the items used to assess the sample apps [20]. Both coders assessed the content of the sample apps independently. Finally, the researchers and the coders discussed disagreements until a consensus was reached [18].

### Data Analysis

Descriptive statistics were calculated for all items under the MARS and PPM. The Cronbach *α* was used to evaluate the reliability between each item under the 5 criteria of the MARS, engagement, functionality, aesthetics, information quality, and subjective quality. The Pearson correlation coefficient was then calculated to determine the relationship between the MARS rating and app user rating. The Cronbach *α* was used to evaluate the reliability between each measure item under the 3 factors of the PPM (predisposing factors, enabling factors, and reinforcing factors) and items used by reviewers to assess the app’s ability to promote health behavior change. Multivariate regression analysis was then performed to test the influence of PPM factors on the app’s ability to promote health behavior change, as assessed by reviewers.

## Results

### mHealth App Sample Selection

**Figure 2:**
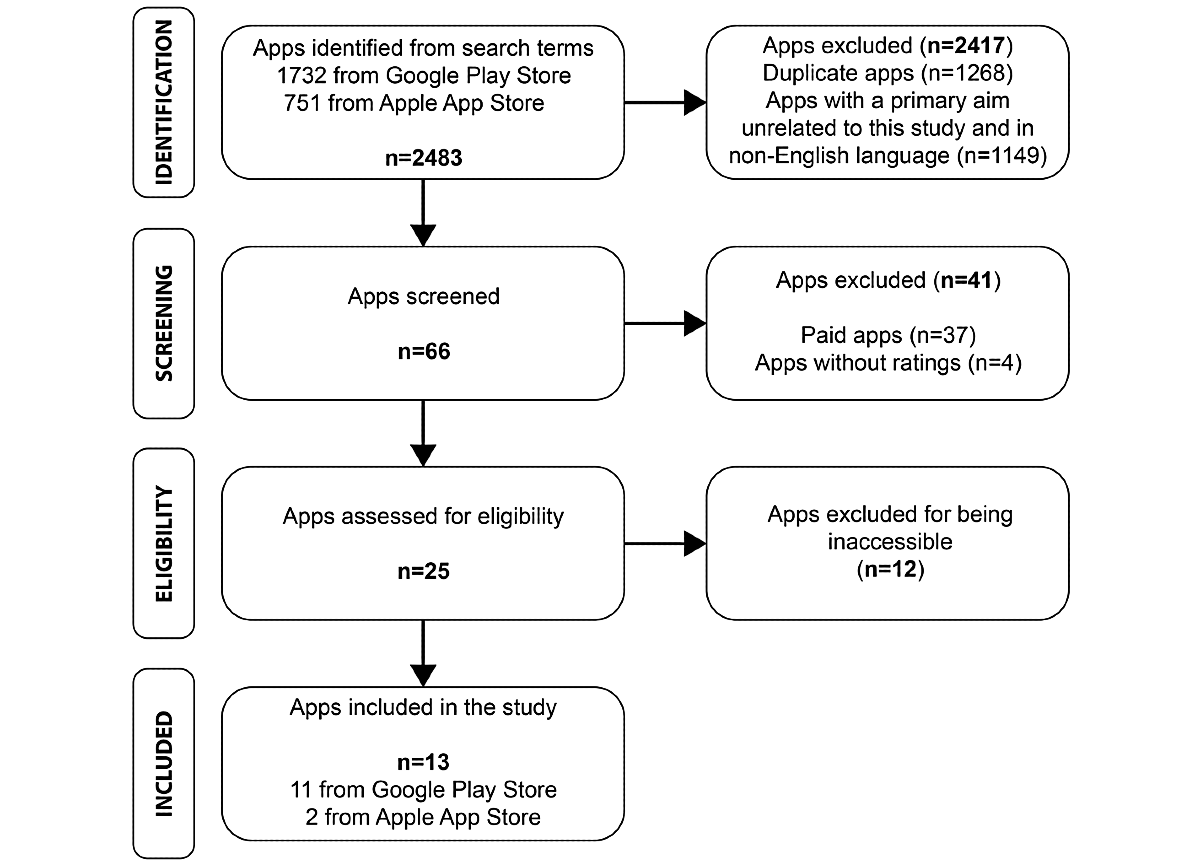
PRISMA (Preferred Reporting Items for Systematic Reviews and Meta-analyses) flowchart of the obesity related mHealth apps selection process.

The initial search with the following search terms resulted in 2483 apps from the Google Play Store (n=1732) and the Apple App Store (n=751): “obesity”, “obese”, “obesity calculator”, “obesity diet”, and “obesity exercise”. Figure 2 shows a flowchart of the obesity-related mHealth app selection process. Descriptive information on the sample apps is presented in Multimedia Appendix 2.

### General Quality: MARS

Among the Google Play Store apps chosen for the study (Table 1), Fitpaa- Your Fitness Dad received the highest score in the engagement (4.6) and information (4.2) categories. The app Fat to Fit – lose weight at home female workout received the highest score in the functionality domain (4.5); Weight Loss Diet 7 Day Detox Cleanse received the highest score in the aesthetics domain (4.3) and Indian Diet Plans received the highest score in the subjective quality (4.0) domain. Among the Apple App Store apps chosen for the study, Jeewith received the highest score in the functionality (3.2), and aesthetics (4.0) domains and IFSO received the highest score in the engagement (2.6), information (3.1), and subjective quality (2.0) domains. Fitpaa – Your fitness dad and Obesity Treatment received the highest overall mean scores based on each dimension of the MARS (3.7).

**Table 1:** The quality of obesity-related mHealth apps based on the Mobile Application Rating Scale.

App Name		Engagement	Functionality	Aesthetics	Information	Subjective quality	Overall score
**Google Play Store apps**							
	Weight Loss Protocols	3.2	4.2	3.0	3.8	3.5	3.5
	Fat to Fit – lose weight at home female workout	4.4	4.5	3.0	3.2	2.5	3.5
	Fitpaa – Your fitness dad	4.6	3.7	3.0	4.2	3.25	3.7
	Lose Belly Fat Guide	2.0	3.5	2.0	1.5	1.0	2.0
	Help for Kids Health and Diet	3.2	3.7	3.0	2.7	3.0	3.1
	Obesity Treatment	3.4	4.0	4.0	4.0	3.2	3.7
	Obesity Guide	1.6	3.7	1.6	1.4	1.0	1.8
	Indian Diet Plans	3.6	4.0	3.6	2.8	4.0	3.6
	Obesity Treatments	2.8	3.0	2.0	2.0	1.5	2.2
	Weight Loss Diet 7 Day Detox Cleanse	3.0	4.2	4.3	2.1	1.7	3.1
	Child Diet Guide	2.6	4.0	2.6	1.8	1.0	2.4
**Apple App Store apps**							
	Jeewith	2.4	3.2	4.0	2.1	1.0	2.5
	IFSO	2.6	3.0	3.6	3.1	2.0	2.8

### MARS Rating Versus User App Rating

The reliability of the dimensions of the MARS scores for the sample apps was found to be strongly consistent (Cronbach *α*=.938). Internal reliability was found to be strong for the subjective quality domain (*α*=.947), good for the aesthetics (*α*=.820) and information (*α*=.888) domains, and fair for the engagement (*α*=.791) domain. Internal reliability was found to be poor for the functionality (*α*=.645) domain, so the performance measure item was removed and after doing so, the internal reliability was found to be good (*α*=.826).

The bivariate Pearson correlation was computed to test the relationship between the MARS rating and user app rating. The results (Table 2) show that the MARS rating and user app rating are not statistically significantly correlated (*R*=0.258; *P*=.39).

**Table 2:** The correlation between the Mobile Application Rating Scale (MARS) rating and user app rating (n=13).

Rating		User app rating	MARS rating
**User app rating**			
	*r*	1	0.258
	*P* value^a^	—^b^	.39
**MARS rating**			
	*r*	0.258	1
	*P* value^a^	.39	—

^a^*P* values are derived from a 2-tailed *t* test.

^b^Not applicable.

### The Presence of PPM Factors

Apart from the causes for obesity listed in the coding sheet (Table 3), there were a few other causes mentioned in the sample apps, which include sleep deprivation, certain medications, a diet with high amounts of simple carbohydrates, biological causes, hormonal causes, and the frequency of eating. Apart from the effects of obesity listed in the coding sheet, there were a few more effects mentioned in the study sample apps, including gall stone formations, gout and gouty arthritis, insulin resistance, Alzheimer disease, social stigmatization, depression among youth, sleep apnea, joint problems, liver disease, infertility, and effects on sperm quality.

**Table 3:** The presence of PRECEDE-PROCEED Model factors within the reviewed (n=13) obesity-related mHealth apps.

Factors, variables, and items					Apps, n (%)
**Predisposing factors**					
	**Knowledge and information**				
		About obesity		6 (46)	
		Genetics^a^		5 (38)	
		Overeating^a^		6 (46)	
		Physical inactivity^a^		5 (38)	
		Social issues^a^		2 (15)	
		Psychological factors^a^		3 (23)	
		Hypothyroidism^a^		2 (15)	
		Type 2 diabetes^b^		6 (46)	
		High blood pressure^b^		5 (38)	
		High cholesterol^b^		3 (23)	
		Stroke^b^		5 (38)	
		Heart attack^b^		5 (38)	
		Cancer^b^		6 (46)	
		What is BMI?		4 (31)	
		Classification of BMI		6 (46)	
		BMI calculator		5 (38)	
	**Attitudes, beliefs, and values**				
		Requires log-in		3 (23)	
		Mentions the sources of information		2 (15)	
		Exercise tips from a physiotherapist		2 (15)	
		Food recommendations from a nutritionist		3 (23)	
	**Confidence and motivation**				
		Color indication to create fear		1 (8)	
		Testimonial		0 (0)	
**Enabling factors**					
	**Teach skills**				
		Walking^c^		3 (23)	
		Swimming^c^		1 (8)	
		Cycling^c^		0 (0)	
		Exercise precaution		1 (8)	
		Diet plan		9 (69)	
	**Provide resources**				
		Food calorie chart		2 (15)	
		Healthy recipes		4 (31)	
		Nutritional breakdown of specific food items		1 (8)	
		Representations of food with images		1 (8)	
		In app^d^		3 (23)	
		External link^d^		0 (0)	
		Image demonstration for exercise		2 (15)	
	**Provide services**				
		Treatment for obesity (surgery)		4 (31)	
	**Track or record behavior**				
		Calorie or food tracker		0 (0)	
		Exercise tracker		3 (23)	
		BMI tracker		3 (23)	
		Weekly or monthly report of calories consumed		0 (0)	
		Weekly or monthly report of exercise progress		0 (0)	
		Goal setting		3 (23)	
		Reminders		1 (8)	
**Reinforcing factors**					
	**Interfacing with social media sites for encouragement**				
		Sharing completion of exercises or weight reduction on social media		2 (15)	
	**Support and encouragement**				
		Community		2 (15)	
		Interaction with health professionals		2 (15)	
		Interaction with a trainer or coach		2 (15)	
		Games		0 (0)	
	**Rewards**				
		Rewards for goal completion		2 (15)	

^a^These items are classified as causes of obesity.

^b^These are effects of obesity.

^c^These are general exercise recommendations.

^d^These are video demonstrations for exercises.

### The Relationship Between PPM Factors and Health Behavior Change

Table 4 presents the internal consistency (Cronbach *α*) of PPM variables and the internal consistency of the measure items under the reviewer’s assessment of the app’s ability to promote health behavior change. All the measure items of PPM factors and the app’s ability to promote health behavior change were found to be internally consistent.

A multivariate regression analysis was performed to test the influence of PPM factors on the app’s ability to promote health behavior change, as assessed by the reviewers. The results from Table 5, Table 6, and Table 7 show that the PPM factors are significant determinants of health behavior change (*F*_3,9_=63.186; *P*=.001). The value of *R*=0.977 indicates a strong positive correlation and *R*^2^=0.955 indicates that 95.5% of the variance in the dependent variable (health behavior change) can be explained by the independent variables (PPM factors).

**Table 4:** The internal consistency of PRECEDE-PROCEED Model (PPM) variables.

PPM factors and variables		Excluded items^a^	Internal consistency of items	Internal consistency of variables
**Predisposing factors**				
	Knowledge and information	None	.938	.911
	Attitudes, beliefs, and values	None	.855	
	Confidence and motivation	Testimonial	Not performed as there is only one item	
**Enabling factors**				
	Teaching skills	Cycling and exercise precaution	.710	.845
	Providing resources	None	.830	
	Providing services	None	Not performed as there is only one item	
	Tracking or recording Behavior		.756	
**Reinforcing factors**				
	Interfacing with social media	None	Not performed as there is only one item	.960
	Support and encouragement	None	.899	
	Rewards	None	Not performed as there is only one item	
**App’s ability to promote health behavior change**				
	Enough information to bring about health behavior change (predisposing factors)	N/A^b^	N/A	.827
	Enough resources to bring about health behavior change (enabling factors)	N/A	N/A	
	Enough support to bring about health behavior change (reinforcing factors)	N/A	N/A	

^a^These items were excluded from analysis as there is no variance in scores between the apps, or the items were deleted.

^b^N/A: not applicable. There are no items associated with these variables.

**Table 5:** Model summary for the regression analysis^a^ between PRECEDE-PROCEED Model factors and the reviewer’s assessment of the app’s ability to promote health behavior change.

Model	*R*	*R^2^*	Adjusted *R*^2^	Standard error of the estimate
1	0.977	0.955	0.940	0.50642

^a^Predictors: constant and reinforcing, predisposing, and enabling factors.

**Table 6:** ANOVA results for the regression analysis^a^ between PRECEDE-PROCEED Model factors and the reviewer’s assessment of the app’s ability to promote health behavior change. All data are based on model 1 from the regression analysis.

	Sum of squares	Degrees of freedom	Mean square	*F* test (*df*)	*P* value
Regression	48.615	3	16.205	63.186 (3)	.001^b^
Residual	2.308	9	.256	N/A^c^	N/A
Total	50.923	12	N/A	N/A	N/A

^a^Dependent variable: reviewer’s assessment of the app’s ability to promote health behavior change.

^b^Predictors: constant and reinforcing, predisposing, and enabling factors.

^c^N/A: not applicable.

**Table 7:** Coefficients from the regression analysis^a^ between PRECEDE-PROCEED Model factors and the reviewer’s assessment of the app’s ability to promote health behavior change. All data are based on model 1 from the regression analysis.

Predictors	Unstandardized coefficients		Standardized coefficients		
	*β*	Standard error	*β*	*t* test (*df*)	*P* value
(Constant)	3.922	0.259		15.165 (12)	.001
Predisposing factors	0.112	0.024	.339	4.649 (12)	.001
Enabling factors	0.257	0.065	.440	3.930 (12)	.003
Reinforcing factors	0.581	0.123	.530	4.746 (12)	.001

^a^Dependent variable: reviewer’s assessment of the app’s ability to promote health behavior change.

## Discussion

### Principal Findings

This study aimed to examine the features and quality of obesity-related mHealth apps using the MARS and assess the presence of factors that promote health behavior change using the PPM. We analyzed a total of 13 obesity-related mHealth apps, 11 from the Google Play Store and 2 from the Apple App Store. The Apple App Store had a much lower number of obesity-related mHealth apps compared to the Google Play Store. Regarding the overall quality of the 13 apps assessed using the MARS, the mean scores ranged from 1.8 to 3.7. This study supports the findings of previous studies that suggest when mHealth apps focus heavily on the functionality domain of the MARS, the performance, ease of use, navigation, and gestural design are compromised [20]. The subjective quality domain of the MARS depends on all 4 domains, engagement, functionality, aesthetics, and information. Among all 4 domains, the apps in this study scored the lowest in information. The information domain comprises accuracy, goals, quality of information, quantity of information, visual information, credibility, and evidence-based information. The absence of sources of information in most of the apps studied affected the credibility score and the evidence-based information score. These findings support the findings of previous studies which established that mHealth apps containing evidence-based information and information from credible sources receive high scores in the information domain of the MARS [26] and mHealth apps that do not include sources of information receive the lowest scores [27]. Among the studied apps, all received moderate mean scores for each of the 4 domains of the MARS, engagement, functionality, aesthetics, and information; this affected the mean score for the subjective quality domain of the study sample apps since the subjective quality domain depends on the other 4 domains of the MARS.

There are many mHealth apps currently available for various health issues; finding an appropriate app among the wide selection for a particular health issue is challenging for users [9,28]. Normally, users select an mHealth app based on ratings and reviews; thus, ratings become key for any app to be downloaded by new users [28,29]. We failed to establish a statistically significant Pearson correlation coefficient between MARS scores and the ratings of study sample apps in the app store. This nonsignificant result may be due to information asymmetry between coders and app users with regard to the app quality attributes. The trustworthiness of apps with few ratings may also be compromised by fake reviews from app developers; this may partly explain the nonsignificant result [30].

Most of the study sample apps were established upon predisposing factors to address obesity, including the following variables: knowledge and information about obesity; attitudes, beliefs, and values; and confidence and motivation. Commonly, mHealth app users will form judgements about apps’ contents by evaluating the information using web-based platforms, especially when they come across unfamiliar information about health conditions, and they use the sources of the information to judge its credibility [31]. Therefore, mentioning the sources of information and ensuring that recommendations of exercise and diet plans are provided by health professionals is important; this was found in only a small number of study sample apps. None of the sample apps had testimonials, but previous studies strongly recommended apps add testimonials or narrative messages that focus on real experiences of users, which can lead to strong emotional arousal among users and are an important factor in promoting health behavior change [32,33].

With regard to enabling factors, the teaching skills variable was found in a number of study sample apps. One of the least common enabling factors among the apps was the ability to track or record behavior, which contradicted a previous study on diabetes management apps [34]. Previous studies found that the tracking facility in mHealth apps proved to be motivating and influenced health behavior change among app users, especially for weight loss [35,36]. Self-tracking of food and exercise helps users set goals and track their achievements [9]. The self-tracking, goal setting, and daily, weekly, or monthly reporting features in mHealth apps were found to be very helpful in bringing about health behavior change [9], but those features were also only found in a small number of study sample apps. One important finding from the study is that 69% (9/13) of the sample obesity-related mHealth apps specified diet plans as a measure to address obesity, but only 23% (n=3) of sample apps included exercise as a recommendation. This finding supports the findings of previous studies that the mHealth apps focus either on physical activity or dieting practices, but not equally on both for weight loss [37].

Reinforcing factors, which include interfacing with social media sites for encouragement, support and encouragement from a community or health professionals, and rewards for goal completion, were found to be present in only 2 apps among the study sample, 1 from the Google Play store and 1 from the Apple App Store. This finding is consistent with the findings of previous studies that only a few mHealth apps allow users to connect the app to external systems or communities, such as social media platforms [18]. Sharing task completion on social media is the most welcomed feature by mHealth app users because they can obtain emotional support and motivation from others [9]. Such mobile features help or guide users to undergo health behavior change by establishing interactions with health professionals, allowing them to gain support from their peer group, and providing them with access to a virtual coach. Past studies have shown that a lack of motivation and social support among mHealth app users reduces the likelihood of health behavior change [38]. This study found that most of the sample mHealth apps did not include reinforcing factors, which are considered vital in bringing about health behavior change among app users.

### Limitations

The findings of this study should be taken into consideration with some limitations. First, the obesity-related mHealth apps used in the analysis were free; analyses including paid apps may produce different results since paid apps are generally given extra care during the development of all aspects of the app. This study is not supported by any funding, which is the reason for the omission of paid versions of obesity-related mHealth apps. Similarly, we were also unable to download and study inaccessible apps, which required log-in credentials from an affiliated health care organization or clinic [39]. Second, the study did not collect data from actual users of the mHealth apps; doing so may result in a better understanding of the influence of the apps’ features on health behavior change. This may also open up a new dimension to this study.

### Conclusion

There are numerous mHealth apps available in the Google Play Store and the Apple App Store to promote health behavior change. Previous studies have shown that mHealth apps are more effective when they are based on scientific theories [18]. This study found that the presence of PPM factors in an mHealth app can greatly influence users’ health behavior change. So, this study suggests that mHealth app developers consider this when developing efficient apps. Also, mHealth app developers should consider providing health information from credible sources and including the sources of the information, which will increase the perceived credibility of the apps among users. Users of mHealth apps vary in gender and age group; so, mHealth app developers should concentrate on providing general health behavior tips that can be used by all gender and age groups or tips for specific gender and age groups. Though there are numerous mHealth apps available, there is a paucity in the involvement of health professionals and health organizations in the development of these apps. Most of the available mHealth apps bypass regulations and nationally recognized health guidelines (R Subramanian, PhD, unpublished data, August 2015). So, we strongly suggest health experts be directly involved in the development of mHealth apps rather than third-party developers [37]. The findings of this study make several contributions to the current literature related to mHealth apps. Future research should include actual mHealth app users to better understand the apps’ effectiveness in bringing about health behavior change.

## Appendix

Multimedia Appendix 1 Coding sheet.

Multimedia Appendix 2 Descriptive information of study sample apps.

## Abbreviations

MARS: Mobile Application Rating Scale
PPM: PRECEDE-PROCEED Model
