# Supplementary material for: The Quality of Indian Obesity-Related mHealth Apps: PRECEDE-PROCEED Model–Based Content Analysis
Source: JMIR Mhealth Uhealth. 2022 May 11;10(5):e15719. doi: 10.2196/15719 (PMC9133986; doi:10.2196/15719)
Supplement: Multimedia Appendix 1 [file mhealth_v10i5e15719_app1.docx]

**Multimedia Appendix 1. Coding sheet.**

**Precede Proceed Model**

| **General App Characteristics** | | | | | | | | | | | | | | |
| --- | --- | --- | --- | --- | --- | --- | --- | --- | --- | --- | --- | --- | --- | --- |
| User rating | | | |  | | | | | | | | | | |
| Downloads | | | |  | | | | | | | | | | |
| Reviews | | | |  | | | | | | | | | | |
| **MARS (Mobile App Rating Scale)**  **(1- Very Low, 2 – Low, 3- Fair, 4- Good, 5-Very Good)** | | | | | | | | | | | | | | |
| **Engagement** | | | | | | | | | | | | | | |
| Entertainment | | | |  | | | | | | | | | | |
| Interest | | | |  | | | | | | | | | | |
| Customization | | | |  | | | | | | | | | | |
| Interactivity | | | |  | | | | | | | | | | |
| Target group | | | |  | | | | | | | | | | |
| **Functionality** | | | | | | | | | | | | | | |
| Performance | | | |  | | | | | | | | | | |
| Ease of use | | | |  | | | | | | | | | | |
| Navigation | | | |  | | | | | | | | | | |
| Gestural design | | | |  | | | | | | | | | | |
| **Aesthetics** | | | | | | | | | | | | | | |
| Layout | | | |  | | | | | | | | | | |
| Graphics | | | |  | | | | | | | | | | |
| Visual appeal: How good does the app look? | | | |  | | | | | | | | | | |
| **Information** | | | | | | | | | | | | | | |
| Accuracy of app description | | | |  | | | | | | | | | | |
| Goals | | | |  | | | | | | | | | | |
| Quality of information | | | |  | | | | | | | | | | |
| Quantity of information | | | |  | | | | | | | | | | |
| Visual information | | | |  | | | | | | | | | | |
| Credibility | | | |  | | | | | | | | | | |
| Evidence base | | | |  | | | | | | | | | | |
| **Subjective quality** | | | | | | | | | | | | | | |
| Would you recommend this app? | | | |  | | | | | | | | | | |
| How many times you would use this app? | | | |  | | | | | | | | | | |
| Would you pay for this app? | | | |  | | | | | | | | | | |
| What is your overall star rating of the app? | | | |  | | | | | | | | | | |
| **Precede Proceed Model**  **(1-Yes, 0- No)** | | | | | | | | | | | | | | |
| **PREDISPOSING FACTOR**  (Predisposing factors are intellectual and emotional givens that tend to make individuals more or less likely to adopt healthful or risk behaviours) | | | | | | | | | | | | | | |
| **Knowledge/Information** | | | | | | | | | | | | | | |
| About Obesity | | | |  | | | | | | | | | | |
| Causes for Obesity | | | | | | | | | | | | | | |
| Genetics | Overeating | | Physical  Inactivity | Social issues | | Psychological  Factors | | | | Hypothyroidism | | | | Others |
|  |  | |  |  | |  | | | |  | | | |  |
| Effects of Obesity | | | | | | | | | | | | | | |
| Type 2 Diabetes | High blood pressure | | High Cholesterol | Stroke | | Heart attack | | | | Cancer | | Others | | |
|  |  | |  |  | |  | | | |  | |  | | |
| What is BMI? | | | |  | | | | | | | | | | |
| Classification of BMI | | | |  | | | | | | | | | | |
| BMI Calculator | | | |  | | | | | | | | | | |
| **Attitude/Belief/Value** | | | | | | | | | | | | | | |
| Requires login | | | |  | | | | | | | | | | |
| Mention the source of information | | | |  | | | | | | | | | | |
| Exercise tips from Psychotherapy | | | |  | | | | | | | | | | |
| Food recommendation as per Nutritionist | | | |  | | | | | | | | | | |
| **Confidence/Motivation** | | | | | | | | | | | | | | |
| Color indication to create fear | | | |  | | | | | | | | | | |
| Testimonial | | | |  | | | | | | | | | | |
| **ENABLING FACTORS** | | | | | | | | | | | | | | |
| **Teach Skills** | | | | | | | | | | | | | | |
| General Exercise Recommendation | | | | | | | | | | | | | | |
| Walking | | Swimming | | | Cycling | | | Others | | | | | | |
|  | |  | | |  | | |  | | | | | | |
| Exercise precaution | | | | |  | | | | | | | | | |
| Diet plan | | | | |  | | | | | | | | | |
| **Provide Resources** | | | | | | | | | | | | | | |
| Food Calorie Chart | | | | |  | | | | | | | | | |
| Healthy recipes | | | | |  | | | | | | | | | |
| Nutritional breakdown of specific food items | | | | |  | | | | | | | | | |
| Represent food with image | | | | |  | | | | | | | | | |
| Video Demonstration for exercise | | | | | In app | | |  | | | | | | |
|  |  |  |  |  | External link | | |  | | | | | | |
| Image demonstration for exercise | | | | |  | | | | | | | | | |
| **Provide Service** | | | | | | | | | | | | | | |
| Treatment for Obesity (Surgery) | | | | |  | | | | | | | | | |
| **Track/Record Behaviour** | | | | | | | | | | | | | | |
| Calories or food tracker | | | | |  | | | | | | | | | |
| Exercise tracker | | | | |  | | | | | | | | | |
| BMI tracker | | | | |  | | | | | | | | | |
| Weekly/monthly report of calories | | | | |  | | | | | | | | | |
| Progress of exercise weekly/monthly report | | | | |  | | | | | | | | | |
| Goal setting | | | | |  | | | | | | | | | |
| Reminder | | | | |  | | | | | | | | | |
| **REINFORCING FACTORS** | | | | | | | | | | | | | | |
| **Interfacing with social media sites for encouragement** | | | | | | | | | | | | | | |
| Sharing completion of exercise or weight reduction in social media | | | | |  | | | | | | | | | |
| **Support/Encouragement** | | | | | | | | | | | | | | |
| Community | | | | |  | | | | | | | | | |
| Interaction with health professionals | | | | |  | | | | | | | | | |
| Interaction with trainer or coach | | | | |  | | | | | | | | | |
| Game | | | | |  | | | | | | | | | |
| **Reward** | | | | | | | | | | | | | | |
| Reward for goal completion | | | | |  | | | | | | | | | |
| Do you think there is enough information in pre-disposing factor to bring health behaviour change? | | | | | **None** | | **Minimal** | | **Moderate** | | **Strong** | | **Very strong** | |
|  |  |  |  |  | **1** | | **2** | | **3** | | **4** | | **5** | |
| Do you think there is enough resources in enabling factor to bring health behaviour change? | | | | | **1** | | **2** | | **3** | | **4** | | **5** | |
| Do you think there is enough support in reinforcing factor to bring health behaviour change? | | | | | **1** | | **2** | | **3** | | **4** | | **5** | |
