# Supplementary figures and images for: The Quality of Indian Obesity-Related mHealth Apps: PRECEDE-PROCEED Model–Based Content Analysis
Source: JMIR Mhealth Uhealth. 2022 May 11;10(5):e15719. doi: 10.2196/15719 (PMC9133986; doi:10.2196/15719)

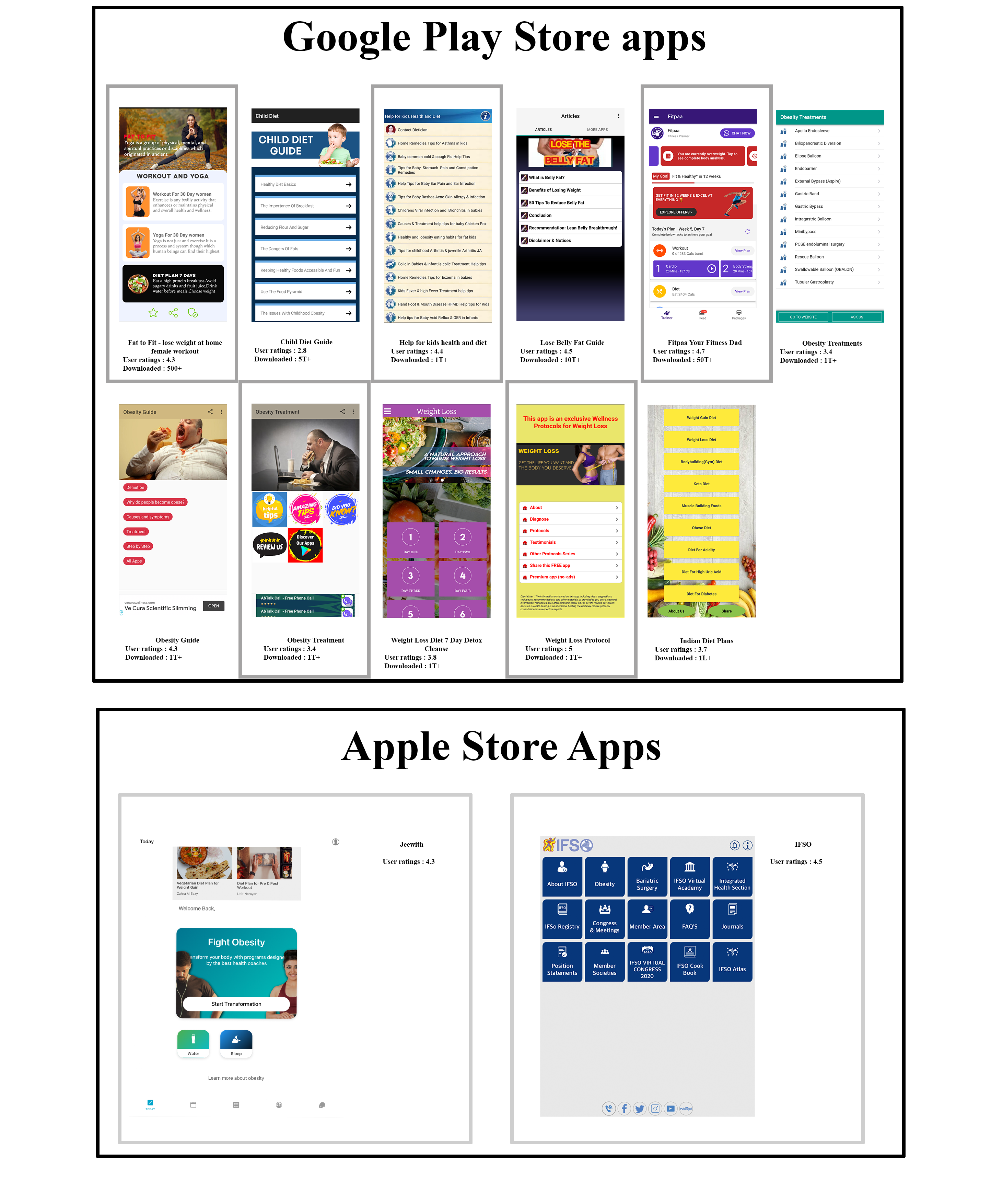

Supplement: Multimedia Appendix 2 [file mhealth_v10i5e15719_app2.png]
